# Insights into Nutritional Strategies in Psoriasis

**DOI:** 10.3390/nu15163528

**Published:** 2023-08-10

**Authors:** Carolina Constantin, Mihaela Surcel, Adriana Munteanu, Monica Neagu

**Affiliations:** 1 Immunology Department, Victor Babes National Institute of Pathology, 050096 Bucharest, Romania; caroconstantin@gmail.com (C.C.); msurcel2002@yahoo.com (M.S.); iadinuta@gmail.com (A.M.); 2 Pathology Department, Colentina Clinical Hospital, 020125 Bucharest, Romania; 3 Doctoral School, Faculty of Biology, University of Bucharest, 050107 Bucharest, Romania

**Keywords:** psoriasis, obesity, nutrition, diet, microbiome

## Abstract

Psoriasis, an autoimmune chronic inflammatory skin condition, has a high incidence in the general population, reaching 2–4%. Its pathogenesis involves an interplay of genetic factors, immune disturbances, and environmental factors. Within the environmental factors that aid the appearance of this autoimmune skin disease, the Western lifestyle and overall diet play important roles in the steady growth in psoriasis prevalence. Furthermore, psoriasis is associated with comorbidities such as psoriatic arthritis, cardiovascular disease, metabolic syndrome, and obesity. Accumulating evidence suggests that obesity is an important risk factor for psoriasis. Moreover, obesity aggravates established psoriasis, and a reduction in the body mass index can improve the clinical outcomes of psoriasis and increase the efficacy of standard psoriasis therapies. The possible connection between this autoimmune disease and obesity relies on the fact that white adipose tissue is an essential endocrine organ that secretes an array of immune mediators and inflammatory and metabolic factors with pro-inflammatory action. Thus, immune-mediated mechanisms in both psoriasis and obesity conditions are common factors. This paper describes the factors that link obesity with skin autoimmune disease and highlights the importance of the stimulatory or regulatory effects of nutrients and food in psoriasis and the possible improvement of psoriasis through nutritional strategies.

## 1. Introduction

Psoriasis (Ps) is a chronic, multi-etiological disease with a profound inflammatory pattern. It is experienced by around 3% of individuals worldwide, affecting mainly adults. Ps is associated with various comorbidities [1] and patients having an overall decreased quality of life [2]. Genetic factors combine with extrinsic factors to initiate psoriatic lesions. Among the extrinsic factors, trauma, infections, various medications, exposure to sunlight, stress, habitual factors (e.g., alcohol, smoking), obesity, and endocrine factors add to the panel of factors that induce and sustain Ps [3].

From a histological point of view, a psoriatic lesion is characterized by the hyper-proliferation of keratinocytes and increased infiltration of immune cells (e.g., T lymphocytes, dendritic cells, and neutrophils). A dermatologist will evaluate the Ps lesion area and severity index (PASI), body surface area (BSA), and dermatology life quality index (DLQI) in order to classify the severity of the disease in a psoriatic patient [4]. According to the recent 2021 classification, Ps patients are classified as having mild, moderate, and severe forms based on the combined use of the PASI, BSA, and sPGA (static Physician Global Assessment). sPGA is performed by the dermatologist to assess erythema, induration, and scaling. A recently published algorithm can identify severe forms in patients that meet two out of three criteria (PASI ≥ 11 or BSA ≥ 10 or sPGA ≥ 3), mild forms in patients that meet two out of three criteria (PASI ≤ 3 or BSA ≤ 5 or sPGA ≤ 2) and have a DLQI of <5, and moderate forms of disease for patients that do not meet two out of three criteria (PASI ≥ 11 or BSA ≥ 10 or sPGA ≥ 3) but have a DLQI of ≥5. This recent classification relies on a ranking algorithm that can aid dermatologists in monitoring the disease’s clinical evolution and establishing a specific therapy for the patient [5].

**Figure 1:**
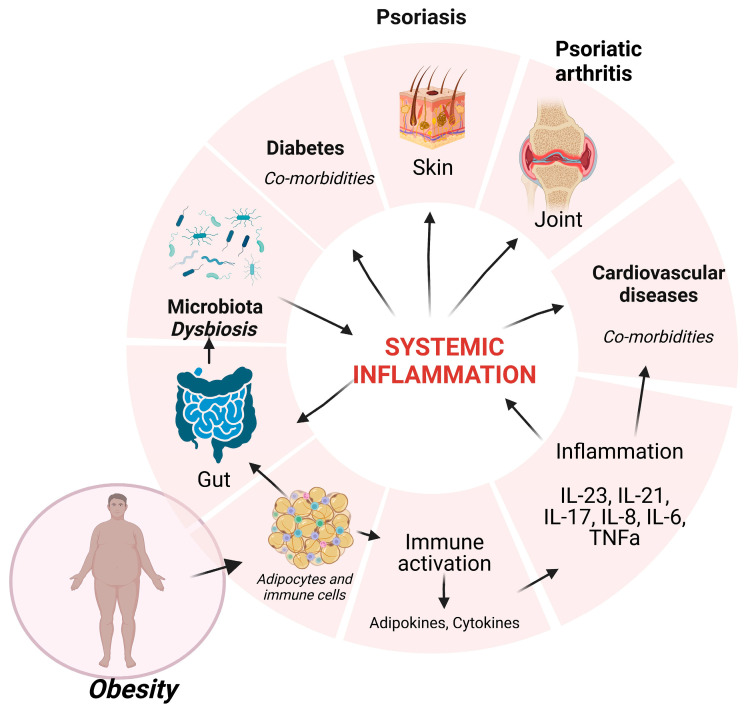
Obesity influences the homeostasis of multiple systems. Adipocytes and immune cells resident in fat tissue induce pro-inflammatory factors that sustain chronic inflammation (IL-23, IL-21, IL-17, IL-8, IL-6, TNFα) that will generate systemic inflammation, inducing psoriatic events and comorbidities (diabetes, cardiovascular diseases). This systemic inflammation will deregulate the gut microbiota, and the induced dysbiosis will contribute to psoriasis. Created with BioRender.

Recently, obesity has been suggested to be one of the factors that clearly influence psoriatic events; the association showed that there is an increased prevalence of obesity in psoriasis patients, compared with the general population [6]. Fatty tissue is a reservoir of endocrine and pro-inflammatory factors that overwhelm the body’s homeostasis and further induce systemic deregulation in multiple organs. A general scheme depicting the factors that are present in obesity and Ps is presented in Figure 1.

Recent studies have evaluated nutritional regimens in psoriatic patients in order to improve the clinical outcomes of the disease. Clinical parameters associated with the metabolic profile and the inflammatory state of patients subjected to various diet regimens were evaluated. A low-calorie diet and a ketogenic diet, among others, improved all the tested parameters and clinically alleviated the psoriatic status of the patients [7,8].

Without being exhaustive, this review paper summarizes recent studies describing the link between Ps and obesity, the microbiota pattern inducing both obesity and autoimmune disease, a complex animal model of Ps and obesity that highlights new molecular mechanisms that govern these processes, and updated nutritional strategies that can contribute new adjuvant therapies for the treatment of psoriatic disease.

## 2. Psoriatic Patients and Obesity

The multifactorial nature of Ps pathogenesis includes metabolic process disruption, among which obesity has been recognized as a novel risk factor with rising importance in Ps onset and severity. Both obesity and Ps are associated with a chronic pro-inflammatory status in which microbiome deregulation has been identified to play a leading role [9].

### 2.1. Obesity in Psoriasis Onset

Obesity is defined as a body mass index (BMI) of ≥30 kg/m^2^, and this metabolic status poses both a significant global health concern and an economic burden [10]. In recent years, accumulating data has connected Ps incidence to excess body weight, as people suffering from Ps are usually overweight in comparison to the rest of the population. Interestingly, in May 2022, the WHO released a complex report that emphasized that obesity prevalence has almost tripled since 1975, and furthermore, over 60% of European citizens are overweight or even obese. The report highlights the cumulative inferences of the COVID-19 pandemic in respect of obesity, with all the repercussions that have resulted in terms of morbidity and mortality [11]. Obesity has thus acquired the roles of both a trigger and a contributor in the development of various diseases in which metabolic deregulation plays an important role, and Ps is one of these diseases. Moreover, a large meta-analysis of observational studies has suggested that obese people suffering from Ps have a higher risk of psoriatic arthritis, with a 6% surge in complications for every 1 kg/m^2^ increase in BMI [12].

Various studies have underlined novel insights regarding the severity of Ps lesions and the risk of obesity. Armstrong et al. explored the link between these two parameters in a meta-analysis that included over 2 million participants, of whom over 200,000 were patients with Ps. The authors showed that in Ps patients, the risk of obesity is over 50% compared to healthy subjects. Going further into the analysis, patients with more serious forms of Ps present a higher risk of obesity compared to patients diagnosed with the mild form. In addition, patients with Ps and normal body weight are prone to becoming obese in the future [13]. Along with more severe psoriatic lesions, psoriatic obese patients can also encounter several metabolic comorbidities (e.g., fatty liver, hyperlipidemia, etc.) that worsen their clinical disease profile. Additionally, an elevated BMI is a significant risk factor for many other disorders with a chronic autoimmune background, such as psoriatic arthritis (PsA) [10]. Moreover, patients with PsA experience serious metabolic- and inflammatory-driven comorbidities such as obesity, hypertension, or diabetes, as well as cardiovascular disease, in comparison with the general healthy population. The inflammatory milieu in PsA is attributed to adipose tissue, whose dysregulation maintains chronic low-grade systemic inflammation [14]. Therefore, in order to reduce the risk of developing metabolic complications, it is mandatory for Ps and PsA patients to control their BMI primarily through losing weight [15]. The most accessible strategy for lowering BMI and reducing adipose tissue in Ps patients is a low-calorie diet. Recently, diet as a potential therapeutic support method in Ps management has been exploited through the on-going Diet and Psoriasis Project, which aims to assess whether dietary factors are related to Ps severity (e.g., time-restricted eating vs. Mediterranean diet) [16].

According to various studies, body composition and body fat distribution are parameters that are more reliable than BMI, reflecting more accurately the patient’s nutritional status, and thus allowing observation of the in-depth correlation between weight and lesion severity, and monitoring the disease course. By using various refined methods that assess the nutritional status of psoriatic patients (e.g., bioimpedance analysis), the analysis of body composition has revealed that Ps is associated with elevated levels of body fat, visceral fat, and a diminished muscle mass [17], and thus bioimpedance analysis is endorsed as having a much better diagnostic power compared to BMI assessment alone [18].

Thus, the obesity–psoriasis–nutrition axis could be explored to decrease inflammation at the gut and skin levels. The result of such a strategy in terms of the disease course could be evaluated by measuring specific markers, such as decreases in C-reactive protein, TNF-α, and IL-6 levels; these parameters have been linked to a diet rich in soluble fibers [19].

### 2.2. Adipose Tissue Dysregulation in Psoriatic Patients

There are two forms of adipose tissue in humans: white tissue, which is the most abundant form and stores energy, and brown tissue, which is found especially in human newborns and plays an important role in regulating body temperature. Adipocytes are the most abundant cell type in white adipose tissue, along with pre-adipocytes (adipocytes that are unloaded yet contain lipids), endothelial cells, fibroblasts, leukocytes, and macrophages. Moreover, the number of macrophages in white adipose tissue has been found to be directly correlated with the degree of obesity [20].

Obesity can also be defined as an overbalance in the ratio between energy intake and energy consumption. As obesity evolves, body fat will excessively accumulate, giving rise to unhealthy white adipose tissue that becomes inflamed [21]. This adipose-related inflammation correlates further with chronic low-grade systemic inflammation. The specific low-inflammatory status is now a well-recognized mechanism for an increased risk of many serious pathologies (e.g., cardiovascular diseases), including those related to skin manifestation as Ps is [22,23].

In obesity, the alteration of adipogenesis is the leading step to the hypertrophy of adipocytes and further inflammation [24]. In this context, the modified adipose tissue will start to release a specific category of bioactive factor known as adipokines, whose types and levels depend on various factors such as the types of activated adipocytes (white or brown cells), number of cells, site, and interaction with other immune and non-immune cells. Moreover, in light of recent studies, adipose tissue should be considered not only a simple energy storage deposit but also a dynamic endocrine organ that actively intercedes in the regulation of inflammation, metabolism, and immunity, as well as in other physiological processes [25].

Studies in the last decade focusing on obesity onset have identified the entire flow of cellular processes involved in normal to hypertrophic adipose tissue transition, resulting in increased systemic inflammation and inhibition of adipogenesis. Excessive, unbalanced nutrition associated with other factors such as stress and/or a lack of physical exercise can lead to hypertrophy of adipocytes and eventually to obesity. Subsequently, mature adipocytes start to secrete free fatty acids and express an important array of pro-inflammatory adipokines (e.g., leptin, resistin, visfatin, chemerin, etc.) [26]. In addition, the expression of anti-inflammatory adiponectin is inhibited, which causes local inflammation and the recruitment of immune cells. There are increased numbers of locally infiltrating macrophages in obese adipose tissue, a process associated with fibrosis that also alters hormonal equilibrium [27]. Moreover, adipocytes and pre-adipocytes secrete a panel of pro-inflammatory cytokines and chemokines, including IL-6, CCL2, IL-1β, and TNF-α, which fuel low-grade systemic inflammation in obese individuals and induce metabolic syndrome [28].

In Ps patients with a severe course of the disease, increased blood levels of pro-inflammatory adipokines have been observed, and during lesion remission, these levels were found to be decreased. Additionally, serum levels of the anti-inflammatory adipokines omentin and adiponectin are significantly lower in patients with severe disease compared to patients with mild forms of Ps [28].

Considering the role of obesity in Ps development, some adipokines have been thoroughly studied in relation to the inflammatory milieu and severity of lesions. Studies from the last decade have claimed a role for adiponectin in the pathogenesis of Ps [29].

Adiponectin levels control a series of cytokines and the immune cellular balance of certain T lymphocyte subsets. Thus, in keratinocytes from Ps lesions, adiponectin inhibits pro-inflammatory cytokine synthesis and increases the release of anti-inflammatory cytokines [30,31]. At a cellular level, this would be translated as a restoration of the Th1-Th17/Th2 lymphocyte ratio, hindering IL-17A synthesis as a key part of adiponectin’s anti-inflammatory effects [32].

Adiponectin plasma levels correlate with a Ps patient’s weight and clinical outcomes. In the plasma of Ps patients, there are lower levels of adiponectin, resulting in an increased pro-inflammatory cytokine pattern and a decreased anti-inflammatory cytokine milieu [33], which may aggravate the severity of skin lesions [12]. Alternatively, with weight loss, the concentration of circulating adiponectin increases, concomitant with psoriatic lesion improvement [34].

Another adipokine, leptin, is involved in regulating the body’s energy balance and weight [35] by raising lipolysis and lowering hepatic lipogenesis [36]. In patients with obesity and psoriatic patients, leptin levels were found to be significantly increased, with a high level of leptin being directly correlated with BMI and with the PASI score [37]. Like adiponectin, leptin is responsible for increasing the pro-inflammatory cytokine pattern, which causes and favors keratinocyte proliferation. In addition, at the cellular level, leptin can impact T helper and dendritic cell functions, thus affecting the immunity processes in Ps [38].

Resistin is another adipokine currently regarded as a potential biomarker in Ps pathogenesis, as it induces a series of molecular and cellular events in psoriatic skin. Resistin is synthesized in adipose tissue mostly by macrophages and monocytes under pro-inflammatory conditions [39]. Further, resistin can induce keratinocyte proliferation via pro-inflammatory factors secreted by B lymphocytes [32]. Impacting the cellular immune network at the skin level, resistin plays an important role in Ps, as resistin can affect both the number and proliferation capacity of Foxp3+ regulatory T (Treg) cells. These deficiencies in Treg cell functionality reinforce the immune-related deregulation in Ps [40]. Prior studies have indicated a direct correlation between Ps severity and plasma resistin levels [41], while elevated plasma resistin levels in patients with Ps were strongly correlated with the *DLQ* index [32].

Chemerin is an adipokine produced mainly in white fat adipocytes, but is also produced by hepatocytes, and plays a role in the pathophysiology of Ps [42,43]. Although the precise function and mechanism of Ps pathogenesis are still in debate, positive correlations between systemic chemerin and obesity-related phenotypes (insulin resistance, BMI, serum triglycerides, etc.) have been registered, suggesting an important function of chemerin in metabolic diseases [44]. Another positive correlation between chemerin levels, inflammatory cytokines, and C-reactive protein was reported in Ps [45,46]. Chemerin acts as a chemotactic factor for human blood plasmacytoid dendritic cells (pDCs), promoting their migration and recruitment in psoriatic skin [47]. Skin exhibiting early and active lesions shows high expression of chemerin in the dermis, while skin exhibiting chronic lesions has low chemerin expression, associated with few pDCs in the dermis. Chemerin expression has been found to be upregulated mainly in fibroblasts linked with higher levels of chemerin mRNA than fibroblasts from healthy or unaffected psoriatic skin. Therefore, chemerin expression specifically labels the early phases of evolving skin psoriatic lesions [48]. The upregulation of chemerin in Ps was assessed in a recent study involving an imiquimod-induced mouse model of Ps. It was observed that intraperitoneal inoculation of an anti-chemerin antibody reduced epidermal proliferation and inflammation in the experimental mice, indicating that chemerin promotes keratinocyte proliferation and enhances the production of inflammatory cytokines, thereby aggravating the Ps. That study proposes chemerin as a forthcoming target for Ps treatment [49].

Other important adipokines, including retinol-binding protein-4, fetuin-A, and lipocalin-2, are being studied as potential mediators in obesity and Ps, although further studies are needed to support this hypothesis [34]. Nevertheless, apart from the cellular and molecular events that cause prolonged inflammation fueled by an array of pro-inflammatory markers, there is another component that impacts the behavior and lifestyle of obese psoriatic patients. Hence, the comorbidity picture of these individuals is completed by depression as a psychological stressor that contributes to the development of anxiety disorders [50,51]. Therefore, understanding the molecular mechanisms of the alterations suffered by adipose tissue in obesity is important because, through the metabolic and functional disorders suffered during the onset of obesity and through maintaining an inflammatory environment, adipose tissue could be analyzed as a potential multifaceted target in the management of obesity in Ps [27].

### 2.3. Microbiota in Obese Psoriatic Patients

Contributing to the multifactorial etiology of Ps, an altered microbiota can be an important trigger in obesity development, as a strong relationship has been documented between the microorganisms that inhabit internal and external body surfaces and are present in autoimmune diseases [52]. Firmicutes, Bacteroides, Proteobacteria, and Actinobacteria represent >98% of the gut microbiota. In respect of Ps, several studies have demonstrated that the relative abundances of Proteobacteria and Bacteroides decrease while the relative abundances of Actinobacteria and Firmicutes increase [53,54]. Like in Ps, in obese subjects, changes in the gut microbiota composition have been registered and significant alterations have been observed in the Firmicutes and Bacteroidota (F/R) ratio compared to normal-weight subjects. The F/B ratio is now broadly recognized as a critical marker in controlling normal gut homeostasis and is an important indicator of the gut microbiota’s status [53]. Variations in F/B lead to dysbiosis, and this ratio was found to be increased in obesity and decreased in some inflammatory conditions, such as Ps [55] and inflammatory bowel disease [56,57]. Some microbiota disturbances at the level of *Clostridium innocuum*, *Eubacterium dolichum*, *Catenibacterium mitsuokai*, *Lactobacillus reuteri*, *Lactobacillus sakei*, and *Actinobacteria* have also been described in obesity, reinforcing gut microbiota dysbiosis as one of the crucial characteristics of this condition [58]. Moreover, in obese psoriatic patients, a state of chronic inflammation is fueled by pro-inflammatory cytokines and adipokines generated by white fat adipocytes and characterized by high levels of IL-17, IL-23, TNFα, and IFNγ, supporting the relation between the microbiome and obesity in psoriatic individuals [59,60,61]. The volume of research in the field that is focused on the comorbidities of Ps is increasing, and recent studies have proved that the intestinal microbiota in patients with obesity suffering from Ps undergoes changes in terms of composition and abundance [62].

## 3. Animal Experimental Models—Direct Proof of the Link between Psoriasis and a High-Fat Diet

### Gut and Skin Microbiome in Animal Models of Psoriasis

Animal models are important tools by which to study skin autoimmune diseases and the mechanisms that underlie their generation [63]. Within the animal models used in this area, imiquimod (IMQ)-induced Ps mice were historically used in the first established model and, hence, are the most commonly used subjects. IMQ is basically a toll-like receptor 7 (TLR-7) agonist that induces skin inflammation in mice, the inflicted skin condition having Ps characteristics [64]. In this type of model, several important clues have been found regarding the skin–gut axis. It was reported several years ago that depletion of the microbiota using antibiotics ameliorated Ps skin inflammation [65], as the microbiota was disturbed by Ps induction [66]. Lactobacillus predominates in the gut microbiota of Ps mice treated with antibiotics [67]. The skin neuroendocrine system has specific activities [68] and regulates several important pathways, such as the inflammatory status and microbiome elements. Skin neuroendocrine system deregulation can cause known inflammatory-mediated pathologies such as Ps, allergies, or atopic dermatitis [69].

Contradictory results have been published regarding the skin microbiota in the Ps-model context, but a recent study has shown that in this model, significant deregulations can be found in the alpha- and beta-types of bacteria comprising the skin’s microbiota. In the gut microbiota, the species *Lactobacillus intestinalis, Lactobacillus reuteri*, and *Bacteroides uniformis* were identified in IMQ mice. Additionally, in the skin microbiota, the species Staphylococcus lentus was identified in IMQ mice. Moreover, correlations between some microbes residing in the intestine and the skin were observed, sustaining the skin–gut–microbiota link in Ps animal models [70]. Lactobacillus intestinalis, Lactobacillus reuteri, and Lactobacillus taiwanensis were found to be significantly higher in the guts of Ps mice [70], and, interestingly, knowing the beneficial actions of Lactobacillus reuteri on the host immune system [71], it seems that in this mouse model, a compensatory immune response was obtained.

In another recent study using a gnotobiotic mouse model of Ps, several new additional clues were identified regarding the skin–gut microbiota in the context of experimental Ps [67]. Previously, it was shown that the presence of intestinal microbiota promotes IMQ-induced skin inflammation and augments the Th17 response [72]. During IMQ-induced skin inflammation, it was shown that there was a decrease in skin microbial diversity. This process was also shown in patients with Ps [73]. Milder skin inflammation in mice is associated with an abundance of *Proteobacteria* and low abundances of *Staphylococci* and *Streptococci* [73].

In another animal model of IMQ-induced Ps, stool samples from healthy donors and Ps patients were used to populate mice’s gut flora. The evolution of Ps in the IMQ-induced mice was aggravated when Ps patient stool was used in comparison to the healthy donor stool. Actually, healthy fecal microbiota protected against the Treg/Th17 imbalance. Sequencing of 16S rRNA showed that *Lactobacillus reuteri* is enriched in the fecal and cutaneous microbiomes of healthy mice compared to Ps mice. Adding *Lactobacillus reuteri* induced the expression of the anti-inflammatory gene IL-10, reducing the Th17 cell populations and inflammation in Ps mice with gut microbiota dysbiosis [74].

With the advent of genetic engineering technologies, several Ps-mouse models have been designed to overexpress or eliminate gene(s) of interest [75]. There are known to be a few global gene knock-in or knockout mouse models developing a Ps-like phenotype (Supplementary Material Table S1) or other genetic designs that modulate mutant alleles [76,77]. Supplementary Material Table S1 presents the main engineered mouse models of Ps [78,79,80,81,82,83,84,85].

Other new animal models involve cell-specific overexpression. The cloning methodology has offered researchers models using KC-specific promoters (keratin [K] 5, K10, K6, K14, involucrin, and loricrin) that overexpress an array of cytokines, adhesion molecules, growth factors, and hormone levels [86,87,88,89,90], leading to inflammation (Supplementary Material Table S2). Moreover, in a K14eIL17Aind/þ mouse model, an increase in circulating IL-17A promotes vascular inflammation, arthritis, and ocular inflammation [89,90].

Other models with mutations in the CARD14 gene have caused Ps and matched the finding that in Ps patients, CARD14 mutations were detected [91]. The Card14E138A/þ (gain-of-function) and Card14DQ136/þ mutations induced in mice spontaneous Ps-like skin inflammation with increases in IL-23/IL-17A cytokine production [88,92]. Moreover, genetically engineered models have been challenged with various pro-inflammatory cytokines to augment skin inflammation. These acute IL-23-induced animal models [93] and Rag2e/e mutated mice [94] provided information regarding various cellular and molecular events within Ps development.

IMQ-induction in, e.g., IL17ra mouse models, has shown the contributions of cell type and gene expression in Ps development [95]. In very new Ps animal models, it was demonstrated that the topical application of small interfering RNA (siRNA) can target genes of interest. The models are evolving, although some cost limitations will hinder their large-scale utilization. Although costly, this approach is more efficient in terms of time–cost parameters compared to the new lines of engineered mouse models [96,97,98].

Animal models have shown that deregulations of the microbiome induced by the Western diet can elicit inflammation and impact the severity of the Ps phenotype [99].

Results obtained from the aforementioned animal models of Ps have shown that gut microbiota dysbiosis influences the development of Ps and therefore it could be a potential therapy target in Ps patients.

## 4. Nutritional Therapeutical Strategies in Psoriasis

### 4.1. Skin–Gut Link—Inflammation Is the Trigger of Deregulation

The notion of an axis that links the gut and the skin, influenced by the microbiome, has gained increased importance in the last few years. The inflammation that resides in the largest human organ, the skin, can induce malfunction of the intestinal barrier and, hence, gut microbiome deregulation and sustained inflammatory mediators/metabolites that contribute to systemic inflammation [53,100,101]. There is cross-talk between the gut microbiota and elements of the immune system, in which the gut microbiome has an important role in the development and regulation of the immune system’s innate and adaptive components [102]. Any disorder of the gut microbiome can trigger an immune response [103]. Inflammation that is both localized and systemic can be induced by microbiota alterations residing on the epithelial surface. In inflammatory bowel disease patients, bacteria deregulation that induces local inflammation leads to mucosal damage and increased permeability of the gut mucosa [104]. This damage increases the pro-inflammatory cytokines (e.g., IL-12, IFN-γ) that will leak into the systemic circulation, spreading the inflammation [104]. While Ps is a skin disorder, it has a clear systemic inflammatory pattern [105] because it induces inflammation in other organs and systems. Moreover, as already stated, Ps is associated with metabolic disorders [106]; hence, Ps patients can have increased BMI, hypertension, hyperlipidemia, type 2 diabetes, coronary artery disease, and so on [107], all of which describe the main Ps comorbidities [28].

In the last few years, abundant studies have researched the link between skin physiological integrity and gastrointestinal health, showing strong cross-talk between the skin and the gut [108,109]. Ramírez-Boscá et al. showed several years ago that bacterial DNA that appends to the gut microbiome can be identified in the peripheral blood of Ps patients [110]. Recent technologies (e.g., next-generation sequencing protocols) have been thoroughly used to investigate the complex intestinal microbiota composition [111].

The notion of a gut–skin axis relies on two equally important players: the microbiome and skin autoimmunity. Ps is aided by the deregulation of the intestinal barrier, an increase in inflammatory mediators, and the systemic effect of bacterial metabolites [53].

The gut microbiome comprises diverse bacterial species, protozoa, viruses, and fungi. These populations are found mainly in the lower gut and are in a symbiotic relationship with the human host [112]. Aerobic species are specific to the small intestine, while anaerobic species are found in the colon [113]. The main bacterial phyla within the gastrointestinal tract are *Firmicutes (Bacillota), Bacteroidota, Actinobacteria*, and *Proteobacteria*. The actual composition depends on the host’s diet, age, and environmental conditions [114]. In addition to diet and lifestyle, genetic predisposition defines gut microbiome individuality [115].

The symbiotic gut microbiome is crucial to intestinal permeability regulation, metabolism, and the functioning of the immune system [116]. It is known that the gut microbiome triggers immuno-protective responses against potential pathogens, having an indirect effect on the immune system. Concomitantly, the gut microbiota can have a direct effect by binding competitively to epithelial cells, inducing immune tolerance to environmental and dietary antigens [117].

The notion of “gut dysbiosis”, representing a composition and biodiversity imbalance, was found to be associated with Ps and its comorbidities, e.g., inflammatory arthritis, inflammatory bowel disease, metabolic syndrome, cardiovascular disease, depression, and obesity [118,119]. As the gut microbiome deregulates skin homeostasis [120], the assertion was also proven to be correct in the opposite case, as many gastrointestinal diseases have skin manifestations [121]. Gut dysbiosis causes negative impacts on skin integrity and function [122,123]. At the molecular level, microbes disrupting the intestinal barrier and skin homeostasis interfere with mucosal immunity components and signaling pathways that regulate epidermal differentiation [124]. As previously mentioned, the metabolites of gut microbes can inflict cutaneous pathology and hinder the immune response [125]. Bacterial metabolites such as p-cresol and phenol synthesized by *Clostridioides difficile* have been proven to be gut dysbiosis biomarkers. These metabolites can enter the blood circulation and accumulate on the skin, causing skin dryness, disrupting the skin’s function as a physical barrier, hindering epidermal differentiation, and affecting keratinization [126]. Innate and adaptive immune arms are also affected by dysbiosis in Ps [127,128,129]. The main immune cell found to be deregulated in Ps is the T cell, where its function and differentiation are altered; these processes have a clear imbalance in Treg and Th17 [67,130].

The molecular link between the microbiome and the immune system is established through the coupling of pathogen-associated molecular patterns (PAMPs) and their receptor pattern recognition receptors (PRRs). In this family of receptors, toll-like receptors (TLRs) and nucleotide-binding oligomerization domain-containing proteins (NODs) are the receptors expressed by a variety of the host’s immune cells. The gut microbiome is the main source of peptide-glycans, molecules that prime innate immune cells via two PRRs (e.g., NOD1, NOD2) expressed by an array of cells, from intestinal epithelial cells such as Paneth cells to immune cells such as macrophages and DCs [131]. Paneth cells, residing at the base of the small intestinal mucosa crypts, have an important role because they secrete key mediators of host–microbe interactions, balancing the colonizing microbiota and the immune protection against enteric pathogens [132]. NODs are also very important, as demonstrated in Nod1- and Nod2-deficient mice, which exhibit a weakened intestinal barrier to microbes and decreased production of antimicrobial peptides, a- and b-defensins, and RegIII-gamma [131]. In humans, individuals that bear mutations in NOD2 have a high susceptibility to Crohn’s disease development [133,134].

Thus, the host’s PRRs interaction with bacterial antigens induces immune system priming by commensal bacteria [128]. In addition to innate immunity, the adaptive arm is influenced by commensal bacteria. Therefore, this interaction balances the effector T cell population and Treg cells. Moreover, B cell activation leads to specific immunoglobulin A production [135]. In a recent animal model of induced Ps, it was demonstrated that gut dysbiosis enhances Th17-induced skin inflammation [67]. Furthermore, the action of Th-17 affects metabolite production and immune cell activation through the IL-23/IL-17 axis signaling pathway, with these processes inducing keratinocyte hyperproliferation [115]. In animal models, it was shown that the gut microbiome and gut dysbiosis induce chronic systemic inflammation due to pro-inflammatory cytokines unbalancing activated effector T cells and increasing epithelial permeability [101,128]. Gut dysfunction and inflammation were observed in Ps patients [53,136] because the specific alterations in the metabolic gut environment were based on the activation of specific PRPs expressed on the gut epithelial cells. Gut permeability increases as inflammatory cytokines like TNF modify the integrity of epithelial cell junctions. This phenomenon activates effector T cells and unbalances Treg cells, and, hence, autoimmune reactions develop. Chronic systemic inflammation is installed, and metabolites, toxins, and bacteria will enter the systemic circulation as the intestinal barrier is hindered [137]. In the circulation, lipoteichoic acid and lipopolysaccharides are shed from the circulating microorganisms, further promoting the pro-inflammatory status [138]. Endotoxin-peptidoglycan superantigens are related to the autoimmune status of Ps. It was shown that Ps patients test positive for toxins from gut bacteria antigens in skin tests. Hence, biomarkers indicating intestinal permeability (e.g., claudin 3 and fatty acid binding protein) can be found to be elevated in Ps patients [138]. If these alterations in the intestinal microbiota are already a certainty in Ps and obesity, the need to assess these changes has accelerated research towards the discovery of biomarkers to evaluate the affected intestinal barrier. Thus, in a recent study highlighting the gut microbiome’s impact on skin health, high levels of claudin-3 and intestinal fatty acid-binding protein as biomarkers of an injured intestinal barrier have been detected in Ps patients [57,139].

### 4.2. Nutritional Strategies—Reducing Obesity and Inflammatory Status Is the Main Target

#### 4.2.1. Low-Calorie Diet

A recent meta-analysis of the studies that link diet to Ps [140,141] has shown that a low-calorie diet is the most important type of diet that induces clear clinical benefits in Ps. Earlier studies have shown that a caloric restriction of 500 kcal below the calculated dietary requirement and comprising 60% carbohydrate, 25% fat, 15% protein, and an exercise level of 40 min at least 4 times per week improves Ps clinical symptoms considerably. After 24 weeks of this regimen, in the diet group, PASI scores of 75 and 50 were achieved by a significant number of patients [142]. New data that evaluated caloric restriction in Ps patients have shown a regression in the lesions after 4 weeks of dieting. Moreover, a significant reduction in the standard parameters was recorded (DLQI, VAS pain, and VAS pruritus), while PASI scores showed a significant 50% reduction [7]. Ps is known to lead to deregulated biochemical parameters in terms of folic acid, vitamin B12, calcium, bilirubin, cortisol, LDL, and total cholesterol [143]. After the diet, all these parameters significantly improved. As high levels of folic acid and vitamin B12 improve the clinical condition in Ps [143], patients subjected to caloric restriction showed increases in these parameters, and their clinical outcomes improved. Hypocalcemia is another risk factor in Ps. The low-calorie diet [7] indicated an increase in calcium. An antioxidant metabolite, bilirubin, is registered in low concentrations in Ps patients [144], and after dieting, its concentration increases. Low cortisol levels are associated with the psoriatic condition, probably related to the stress that is experienced by these patients [145], and after dieting, the cortisol levels increased [144].

As there are several co-morbidities associated with the Ps condition, the alleviation of these comorbidities has also been studied in the low-calorie regimen. Hence, Ps is associated with cardiovascular diseases. Peripheral arterial tonometry, plasma markers of endothelial function, and standard cardiovascular parameters were evaluated in Ps patients. A low-calorie diet (e.g., 800–1000 kcal/day for 8 weeks) continued for 8 weeks at 1200 kcal/day in Ps patients induced significant reductions in blood pressure and resting heart rate, along with the normalization of several other biochemical parameters (total cholesterol, very-low-density lipoprotein cholesterol, triglycerides, plasma glucose, glycated hemoglobin, and tissue plasminogen activator inhibitor) [146].

The Mediterranean diet, as opposed to the Western diet, was also studied in the Greek population and linked to Ps. A study published in 2019 showed a decrease in PASI scores when patients had a diet rich in vegetables, fish, and extra virgin olive oil, whereas there was an increase in the PASI when the diet was rich in dairy products [147].

#### 4.2.2. Gluten-Free Diet

A gluten-free diet represents avoidance of this protein found in wheat, barley, and rye, with a benefit for Ps patients [148]. Interestingly, gluten intake increased the risk of Ps in patients already diagnosed with celiac disease [149]. Moreover, IgA antigliadin (AGA), which is an antibody common for celiac disease, was also found to be increased in Ps patients [150].

Over 20 years ago, positive results were published in Ps patients consuming a gluten-free diet for 3 months, significantly improving their PASI score [151]. The same group has shown that AGA-positive patients subjected to a gluten-free diet had a reduction in Ki67-positive cells in their psoriatic lesions [152,153], meaning a less proliferative status of the lesions. Other groups confirmed that one year of a gluten-free diet improved the PASI score in groups that had high levels of IgA against gliadin peptides [154].

#### 4.2.3. Supplements

Fish oil has been involved in many supplements that Ps patients use in order to alleviate their symptoms. In 2014, a meta-analysis of several studies regarding fish oil supplementation in Ps observed that while some studies have shown moderate results, others have not found any relevance [155]. Recently, in a large cohort of individuals (over 25,000 enrolled subjects of both sexes), vitamin D (2000 IU/day) and/or omega-3 fatty acids (1000 mg/day) were supplemented in their diet, and the group was followed up for 5 years, registering Ps incidence among other autoimmune diseases. The results of the study show that in the supplemented group, the incidence of autoimmune diseases, including Ps, was reduced by over 22% [156].

Herbal products were also found to be useful in Chinese Ps patients, with improvements in their clinical parameters [157]. The obtained results are difficult to interpret in order to draw a conclusion, as there were various plants and preparations. Out of all of them, it seems that a herbal supplement from *Tripterygium wilfordii* was effective as an addition to conventional therapy (cyclosporine and acitretin), leading to an increased percentage of patients achieving PASI-60 [158].

## 5. Discussion

Ps is a worldwide health issue impacting the quality of life of over 60% of diagnosed patients, which mainly includes those with moderate to severe forms of Ps [159,160]. Ps patients experience higher rates of comorbidities, with metabolic syndrome included [161]. In general, Ps has two age peaks of onset: One between 20 and 30 years of age and the second between 50 and 60 years [162]. In Western countries, both age groups are stricken by metabolic deregulation, including obesity. Ps has many triggers, and it is still unclear whether obesity triggers Ps or vice versa. There are arguments for both cases. Thus, the incidence of Ps in obese individuals is high, and Ps patients can gain weight. However, normalizing Ps patient weight will have a major positive impact on the disease. Moreover, weight loss can have a positive impact on classical Ps medications, improving their efficacy. The common denominator of Ps and obesity is the inflammatory process; therefore, all means that reduce the chronic inflammatory status will improve Ps outcomes. Within the reduction of inflammation in Ps patients, regulating gut dysbiosis through diet and nutrients remains an important tool. Several types of diets and supplements have been evaluated in relation to obesity and Ps. Out of all of the tested regimens, a low-calorie diet is the most important type of diet that induces clear clinical benefits in Ps. As Ps is known to be an autoimmune inflammatory disease, the key phrase used in the dieting and/or diet supplementation of patients is “reduction of systemic inflammation”. The reduction in systemic inflammation, obesity reduction, and dysbiosis regulation will reflect directly on disease severity.

## 6. Conclusions

In recent years, we have witnessed an increasing array of studies actively investigating the relationship between obesity, the gut microbiome, and Ps. As gut microbes may influence the pathogenesis of Ps, the microbiome composition, diversity, and relative abundance in Ps individuals strengthen the gut–skin axis. Gut dysbiosis deregulates the epithelial barrier; therefore, microbial metabolites that circulate outside the gut epithelial barrier can cause systemic inflammation. Gut–skin axis functionality can induce an inflammatory effect due to a gut microbiome imbalance [57]. Modulating the gut microbiome and systemic chronic inflammation can improve the symptoms of Ps. Of the methods that can normalize the systemic inflammation recognized in Ps, diet and supplements can actively improve Ps symptomatology and, hence, the patients’ quality of life.

## Data Availability

The data presented in this study are available on request from the corresponding author.

## Supplementary Materials

The following supporting information can be downloaded at https://www.mdpi.com/article/10.3390/nu15163528/s1, Table S1: Recent genetically engineered mouse models of Ps; Table S2: Ps animal model that over-expresses keratinocyte-cell-specific promoters.
