# Supplementary material for: Insights into Nutritional Strategies in Psoriasis
Source: Nutrients. 2023 Aug 10;15(16):3528. doi: 10.3390/nu15163528 (PMC10458768; doi:10.3390/nu15163528)
Supplement: Supplementary file 1 [file nutrients-15-03528-s001.zip › nutrients-2507549-supplementary.pdf]

nutrients

Supplementary Materials

Insights in Nutritional Strategies in Psoriasis

Carolina Constantin [1,2], Mihaela Surcel [1], Adriana Munteanu [1] and Monica Neagu [1,2,3,*]

Table S1. Recent genetically engineered mouse models of Ps.

Animal Model	Gene	Strain
IL-19–/–	*Il19*	C57BL/6
IL20RB–/–	*Il20rb*	C57BL/6
CD11c-Ecaddel	*Cdh1*	C57BL/6
LATΔCD4	*Slc7a5*	C57BL/6
LATΔRλT	*Slc7a5*	C57BL/6
LATΔK5	*Slc7a5*	C57BL/6
PARP1–/–	*Parp1*	C57BL/6
BKS.Cg− + Leprdb/+ Leprdb (db/db)	*Lepr*	BKS
AhRcVECs-KO	*Ahr*	C57BL/6
icIL-1Ra1 –/–	*IcIl1rn1*	C57BL/6
IL-1RaΔM	*Il1rn*	C57BL/6
Nrf2–/–	*Nfe2l2*	C57BL/6
Raptor-f/f; CD2-cre (Raptor KO)	*Raptor*	C57BL/6
Rictor-f/f; CD2-cre (Rictor KO)	*Rictor*	C57BL/6
S1pr4–/–	*S1pr4*	C57BL/6
Krt14Cre/+-hmgb1f/f	*Hmgb1*	C57BL/6
Il36rΔK	*Il1rl2*	C57BL/6
Il36r–/–	*Il1rl2*	C57BL/6
Trpc4–/–	*Trpc4*	C57BL/6
KOB1–/–	*Bdkrb1*	C57BL/6
KOB2–/–	*Bdkrb2*	C57BL/6
KOB1B2–/–	*Bdkrb1Bdkrb2*	C57BL/6
IRF5–/–	*Irf5*	C57BL/6
miR-205-5p agomiR	*miR-205-5p*	BALB/c
C3–/–	*C3*	C57BL/6
IL36rn–/–	*Il36rn*	C57BL/6
Ovol–/–	*Ovol1*	C57BL/6
humanized DITRA mice	*IL36RN*	
Trim21 siRNA	*Trim21*	BALB/c
TG2–/–	*Tg2*	BC57BL/6
Sirt2–/–	*Sirt2*	C57BL/6N
IL-33–/–	*Il33*	C57BL/6
St2–/–	*St2*	C57BL/6
IL-33f/fK5Cre	*Il33*	C57BL/6
K17 siRNA	*K17*	BALB/c
Csf-1RiCreALK3fl/fl	*CSF1R-ALK3*	C57BL/6
S100a8–/–	*S100a8*	C57BL/6

S100a9–/–	*S100a9*	C57BL/6
R26Cyp1a1	*Cyp1a1*	C57BL/6
Ahr–/–	*Ahr*	C57BL/6
K14creMalt1flfl	*Malt1*	C57BL/6
Zdhhc2–/–	*Zdhhc2*	C57BL/6
C6st-1–/–	*Chst3*	C57BL/6
CD19cre-Itga4flox/flox	*Itga4*	C57BL/6
hCD20TAMCreItga4flox/flox	*Itga4*	C57BL/6
hCD20TAMCreIl10flox/flox	*Il10*	C57BL/6
Mapk8–/–	*Mapk8*	C57BL/6
LysMCre Mapk8fl/fl, Mapk8∂M	*LYSMD4-MAPK8*	C57BL/6
ItgaxCre Mapk8fl/fl, Mapk8∂DC	*ITGAX-MAPK8*	C57BL/6
K14cre Mapk8flfl, Mapk8∂Ep	*KRT14-MAPK8*	C57BL/6
PLA2G4B siRNA	*Pla2g4b*	C57BL/6
LRG–/–	*Lrg1*	C57BL/6
CCR4–/–	*Ccr4*	C57BL/6
c-Jun△/△Mx1-Cre	*cjun*	C57BL/6
c-Jun△/△Cd11c-Cre	*cjun*	C57BL/6
c-Jun△/△K5ERT2-Cre	*cjun*	C57BL/6
Tlr7–/–	*Tlr7*	C57BL/6
Ccl2–/–	*Ccl2*	C57BL/6
Nedd4l–/–	*Nedd4l*	BALB/c
Nedd4lf/f Krt14Cre	*Nedd4l*	BC57BL/6
ApoE/SKH-hr2	*Apoe/Skh-hr2*	C57Bl/6 and BALB/c
sh-EZH2	*Ezh2*	BALB/c
N4BP1–/–	*N4bp1*	C57BL/6
ΔCtermut/mut	*Il22ra1*	B6D2x C57BL/6
Il22ra1–/–	*Il22ra1*	C57BL/6
OFA RAT	*Dsg-4*	Sprague-Dawley RAT
miR-149	*miR-149*	C57BL/6
SRSF10	*mSRSF10*	BALB/c
E130307A14-Rik	*E130307A14-Rik*	BALB/c
Rag2–/– + shCDK7 CD4+ T cells	*Rag2, Cdk7*	C57BL/6

Table S2. Ps animal model that over-express keratinocyte cell specific promoters.

**Molecule Type**	**Name**
Cytokines	IL6, TNFA, IL1a, IFNγ, IL12b, IL36a, IL36a/IL36rn, IL20, Il23
Adhesion molecules	ITGB1, ITGA2-IGAB1, ITGA5-ITGAB1
Growth factors	KGF, TGFB1, Tgfb1, BMP6, AREG, Vegfa, Tgfa, Angptl6
Signaling molecules and transcription factors	MAP2K1, Stat3c, Rac1v12, Mek1
Hormones	Leptin
